# Correlation between moral distress and clinical competence in COVID-19 ICU nurses

**DOI:** 10.1186/s12912-023-01277-x

**Published:** 2023-04-07

**Authors:** Zohreh Kalani, Maasoumeh Barkhordari-Sharifabad, Niloufar Chehelmard

**Affiliations:** 1 Department of Nursing, Research Center for Nursing and Midwifery Care, Shahid Sadoughi University of Medical Sciences, Yazd, Iran; 2 Department of Nursing, School of Medical Sciences, Yazd Branch, Islamic Azad University, Yazd, Iran; 3 Shahid Rahnemoun Hospital, Shahid Sadoughi University of Medical Sciences, Yazd, Iran

**Keywords:** Moral distress, Clinical competence, COVID-19, Intensive care units, Nurse

## Abstract

**Background:**

Nurses’ clinical competence is one of the fundamental necessities for providing safe and effective care. Moral distress, as one type of occupational stressors, can affect various aspects of clinical competence, especially under conditions of complicated medical settings such as the coronavirus disease 2019 (COVID-19) epidemic. This study was conducted with the aim of determining the relationship between moral distress and clinical competence in nurses working in COVID-19 intensive care units (ICUs).

**Methods:**

The study was a cross-sectional study. A total of 194 nurses working in COVID-19 ICU affiliated to Shahid Sadoughi University of Medical Sciences, Yazd, central Iran, participated in the study. Data were collected using Demographic Information Questionnaire, Moral Distress Scale, and Clinical Competence Checklist. Data were analyzed with SPSS20 using descriptive and analytical statistics.

**Results:**

The mean score of moral distress, clinical competence, and skills application were 1.79 ± 0/68, 65.16 ± 15.38, and 145.10 ± 38.20, respectively. Based on Pearson correlation coefficient, there was an inverse and significant relationship between the moral distress score and its dimensions with clinical competence and skills application (P < 0.001). Moral distress was a significant negative predictor that accounted for 17.9% of the variance in clinical competence (R^2^ = 0.179, P < 0.001) and 16% of the variance in utilization of clinical competence (R^2^ = 0.160, P < 0.001).

**Conclusion:**

Considering the relationship between moral distress, clinical competence and skills application, to maintain the quality of nursing services, nursing managers can strengthen clinical competence and skills application by using strategies to deal with and reduce moral distress in nurses, especially in critical situations.

## Background

Nurses are one of the main members of the healthcare system and play a prominent role in providing high quality services and improving the community health [1]. Clinical competence of nurses is one of the fundamental necessities for providing safe and effective care [2], and one of the important factors in providing nursing care is based on standards of professional practice [3]. Clinical competence is a continuous process of acquiring knowledge, attitudes, and skills that bring about creativity and innovation in nursing practice [4], which includes both general competencies and specific competencies [5]. Clinical competence is the preference and target of the nursing profession, and quality care depends on it [6]. Nurses’ clinical competence should be regularly evaluated due to continuous changes in therapeutic environments [2], and positive actions should be taken to promote the utilization of their clinical competence [7].

During the coronavirus disease 2019 (COVID-19) pandemic worldwide, healthcare staff faced challenges due to increased demands and changes in work commitments [8–11]. Many health care personnel were required to increase their working hours due to staff limitations and increased patient load [12]. These changes in the workplace induced by epidemics increase the amount of physical and mental stress of the treatment staff [12]. Recent research reveals the impact of COVID-19 on healthcare staff’s emotional stress [13], depression [14, 15], sleep quality [14, 16], occupational burnout [17, 18], and anxiety [14, 16]. McVicar’s study showed that emergency cases, high workload, lack of human resources, lack of support, weak working relationships, and the need to communicate with patients and relatives were among the main sources of stress for nurses [19]. Some scholars believe that occupational stress affects various aspects of clinical competence, such as effective communication, accountability, responsibility, and ethical and professional norms [20]. Moral distress is one type of stressor that has been much debated in literatures.

Moral distress is known as a complex phenomenon of human experience that arises from the feeling unwillingly involved in an unethical act, with little ability to behave differently or change the situation [21–24]. In moral distress, the actor is aware of a morally correct action, but due to institutional, social or procedural limitations, he/she is unable to perform that morally correct action [25]. Health care staff can experience moral distress when constrained by resource limitations, organizational protocols, or governmental requirements [26–28]. The COVID-19 pandemic has created different situations for incidence of moral distress such as preventing family members from visiting patients, inability to rest, compulsory decision-making to support life, lack of resources such as ventilators, less useful treatments, and the inability to perform normal functions [8, 29–32]. Vig [32] categorized factors causing moral distress during the COVID-19 era as patient-related factors (e.g. less than optimal care due to patient loads), clinical factors (e.g. insufficient medical knowledge of situations allocated), organizational factors (such as pressure to use untested treatments) and policy-related factors (such as frequent guideline changes). These factors have been confirmed globally in studies conducted in France [33]. Norway [34], the Netherlands [10, 35], the United States [36–38], Canada [18], Australia and Italy [39], and the UK [40].

Studies have identified that in addition to individual related factors such as clinical experience [41], age [42], salary [42], work environment [43], and employment status [42], other factors such as job stress [44–48] also affect clinical competence. Moral distress is known as an element of job stress in nurses [49]. The relationship between job stress and clinical competence has been shown in various studies, and investigators have reported contradictory results regarding the relationship between these two variables [44–48, 50]. Job stress has been introduced as an intervening factor that is necessary in the emergence and provision of nursing skills and clinical competence [45].

Maintaining the quality of healthcare services and nursing care is especially important in crises, and identifying the components and factors affecting clinical competence and its promotion has always been the focus of educational, health, and medical systems; also, moral distress is one of the common topics debated in nursing, especially in the COVID-19 pandemic, which can have different effects on the health and performance of nurses, patients, and healthcare organizations. Thus, this research was conducted with the aim of determining the relationship between moral distress and clinical competence of nurses working in COVID-19 intensive care units (ICUs).

## Methods

### Research design

This cross-sectional study was conducted during September to November 2021.

### Participants

The research population consisted of nurses working in COVID-19 ICUs in six teaching hospitals affiliated to Shahid Sadoughi University of Medical Sciences in Yazd, central Iran. These hospitals were the major therapeutic and educational centers, and as general hospitals, offered a variety of specialized services to individuals. Most of the COVID-19 services in Yazd/Iran are provided by these hospitals, and these hospitals were COVID-19 admission centers. It should be noted that patients from southern cities of Iran refer to these hospitals for treatment.

The sample size was calculated by the following formula to explore correlation between moral distress and clinical competence:

$$N={\left[\frac{{Z}_{\alpha }+ {Z}_{\beta }}{c}\right]}^{2}+3$$

Wherein N is the desired sample size, $${Z}_{\alpha }$$ is the standard normal score of 95% of confidence interval = 1.96, $${Z}_{\beta }$$= statistical power at 80%, which is 0.84 and $$c=0$$$$0.5\times Ln\left[(1+r)(1-r)\right]$$ with the Pearson correlation coefficient equaling − 0.2 based on the pilot study. To estimate the sample size, a pilot study was conducted with convenience sample of 30 selected eligible nurses. Data from the pilot study were not included in the final analysis. Due to the possibility of subject attrition, the electronic version of the questionnaire was sent to 200 participants. Nurses who met the inclusion criteria were selected using convenience sampling. The inclusion criteria were: having at least a bachelor’s degree in nursing, having at least 2 months of work experience in COVID-19 ICU [51], informed consent to participate in the study, and access to WhatsApp or Telegram virtual networks. The exclusion criteria were: part-time presence in COVID-19 ICU, work leave or sick leave of nurses at the time of the study, and loss of first and second degree relatives due to infection with COVID-19 in the past 6 months.

Having approved the study proposal and having obtained code of ethics from Ethics Committee of the university, the electronic version of the questionnaires was sent to all the qualified nurses in cyber space and they were also informed through short message service short message service (SMS). Nurses were required to fill out the questionnaires in a comfortable and relaxed environment, preferably at home, and contact the researcher if they had any questions about the completion of the questionnaires.

### Data gathering tools

The data collection tool included three questionnaires: Demographic Information Questionnaire, Moral Distress Scale of ICU Nurses, and Clinical Competence Questionnaire.

Demographic information questionnaire included personal and occupational characteristics such as age, gender, marital status, education level, and work experience in the nursing profession, work experience in COVID-19 ICU, employment status, and work shift.

The second questionnaire was Moral Distress Scale of ICU Nurses, which was developed and psychometrically evaluated by Atashzadeh Shourideh et al. (2012). This tool contains 30 items in three dimensions of improper allocation of responsibilities and powers (10 items), errors (10 items), and violation of ethical principles (10 items). A 4-point Likert scale was used to score this tool in all items [not at all (0) to very much (4)]. The moral distress score was obtained from the average of sum of scores of the items. The face, content, construct, and convergent validity of this scale has been confirmed. The minimum validity index of the items of this scale was 0.85 and the scale content validity index/average (S-CVI/Ave) was equal to 0.94 [52]. The reliability of the whole scale was equal to 0.96 using Cronbach’s α method, reliability of its dimensions was between 0.92 and 0.96, and the test-retest correlation coefficient after a two-week interval was equal to 0.91 [53]. In this study, Cronbach’s α coefficient was 0.84 for moral distress scale.

The third questionnaire was the Clinical Competence Questionnaire, which was developed by Meretoja [54]. The nurses using this questionnaire, not only determine the level of their own competence, but also the frequency of using each skill in clinical practice. This questionnaire measures 73 nursing skills in seven areas: Patient assistance (7 skills), education and guidance (16 skills), diagnostic measures (7 skills), managerial abilities (8 skills), therapeutic interventions (10 skills), quality assurance (6 skills), and occupational and organizational duties (19 skills). Nurses evaluate their competence in each skill on a scale of zero (the lowest possible clinical competence) to 100 (the highest possible competence). Finally, the average overall competence and the average competence of each domain are determined based on a score of zero to 100.

In addition, nurses are asked to determine the frequency of using each skill by marking a 4-point Likert scale in such a way that zero means not using that skill and three means frequent use of that skill. Thus, the range of scores is from zero to 219. Since the number of items in each dimension is not the same, scores based on 100 were also calculated for a better interpretation of the data. The internal consistency coefficient of the seven domains was reported to range between 0.79 and 0.91 in the study by Meretoja et al. [54]. This questionnaire was translated into Persian and validated by Bahraini et al. The validity of the Persian version of the instrument has been confirmed by Bahraini et al. using content validity by expert and nursing faculty clinician’ opinions. Its reliability has been reported to range between 0.75 and 0.89 in seven domains using Cronbach’s α coefficient [3]. The Cronbach’s α correlation coefficient of this instrument was obtained as 0.79 in the present study.

### Data analysis

The data were collected, coded, and imported into SPSS 20. The frequency percentage, mean, and standard deviation were used to describe the demographics, moral distress, and clinical competence. In addition, the independent t-test and one-way analysis of variance (ANOVA) were used to identify the significance of the difference between the means of the groups, while the Pearson’s correlation coefficient was used to identify the level of correlation among the variables. A multiple regression analysis was conducted to predict factors that could affect the clinical competence of nurses. In all cases, P < 0.05 was considered to be statistically significant. Before performing the tests, the normal distribution of the data was checked using Kolmogorov-Smirnov (KS) test (P > 0.05).

### Findings

In the present study, 194 nurses completed the questionnaires. The average age of the participants was 32.48 ± 7.00 years. The participants had an average of 9.49 ± 6.47 years of nursing work experience and 7.70 ± 4.84 months of COVID-19 ICU work experience. As it is observed in Tables 1 and 60.3% were female, 67.5% married, 92.3% had a bachelor’s degree, 32.5% had definitive official employment status, and 88.1% had rotational work shift.

**Table 1:** Demographic characteristics of the participants

Variable		Frequency (%)
**Gender**	Male	77 (39.7)
	Female	117 (60.3)
**Marital Status**	Married	131 (67.5)
	Single	63 (32.5)
**Educational level**	BS	179 (92.3)
	BSc	15 (7.7)
**Employment status**	Fulfilling project	49 (25.3)
	Tentative formal employment	22 (11.3)
	Definitive formal employment	63 (32.5)
	Employment with contract	60 (30.9)
**Type of work shift**	Fixed shift	23 (11.9)
	Rotational shift	171 (88.1)

According to Table 2, the average score of moral distress in the research units was 1.79 ± 0.68. The highest average was related to the dimension of errors (1.86 ± 0.76), and the lowest average was related to Violation of ethical codes (1.69 ± 0.77).

**Table 2:** The mean scores of moral distress and its dimensions

Variables	Mean ± SD
Inappropriate allocation of responsibilities and duties	1.82 ± 0.81
Errors	1.86 ± 0.76
Violation of ethical codes	1.69 ± 0.77
Total score of moral distress	1.79 ± 0.68

The average total score for clinical competence and skills application were 65.16 ± 15.38 and 145.10 ± 38.20, respectively. The highest average was related to the dimension of managerial abilities and the lowest average was related to the dimension of quality assurance (Table 3).

**Table 3:** The mean scores of clinical competence and its dimensions

Variables	Clinical competence	Utilization of clinical competence	
	Mean ± SD	Mean ± SD	Mean ± SD (on the base of 100)
Patient assistance & help	63.16 ± 16.03	13.25 ± 4.30	63.10 ± 20.48
Education and guidance	63.87 ± 17.34	30.17 ± 10.83	62.86 ± 22.58
Diagnostic measures	67.34 ± 16.67	14.43 ± 3.96	68.72 ± 18.87
Managerial abilities	69.92 ± 16.31	17.65 ± 4.49	73.58 ± 18.73
Therapeutic interventions	65.27 ± 17.21	20.19 ± 5.87	67.31 ± 19.57
Quality assurance	60.16 ± 18.30	10.66 ± 3.99	59.24 ± 22.20
Occupational & organizational duties	65.68 ± 16.71	38.72 ± 10.42	67.93 ± 18.28
Total score	65.16 ± 15.38	145.10 ± 38.20	66.25 ± 17.44

According to Pearson’s correlation test, there was an inverse and significant correlation between the average score of moral distress and its dimensions with the average score of clinical competence and the average score of skills application (P < 0.001) (Table 4).

**Table 4:** Correlation coefficients of research variables (Pearson correlation test)

Variable	Clinical competence		Utilization of clinical competence	
	r	P	r	P
Inappropriate allocation of responsibilities and competencies	− 0.33^**^	< 0.001	− 0.31^**^	< 0.001
Errors	− 0.29^**^	< 0.001	− 0.36^**^	< 0.001
Violation of ethical codes	− 0.35^**^	< 0.001	− 0.40^**^	< 0.001
Total moral distress	− 0.37^**^	< 0.001	− 0.41^**^	< 0.001

^**^Correlation is significant at the 0.01 level (2-tailed).

There was a statistically significant relationship between clinical competence with gender (P = 0.003), type of work shift (P = 0.02), nursing work experience (P = 0.04), and COVID-19 ICU work experience (P < 0.001). This relationship between the skills application and gender (P = 0.002), type of work shift (P = 0.01), age (P = 0.03), and COVID-19 ICU work experience (P < 0.001) was also significant.

Using regression analysis, changes in dependent variables (clinical competence and utilization of clinical competence) induced by independent variables (moral distress and demographic characteristics) were predicted. Among the demographic characteristics, the mentioned significant or correlated factors were included in the regression analysis. The regression analysis demonstrated four (moral distress, COVID-19 ICU work experience, nursing work experience, and gender) and five (moral distress, COVID-19 ICU work experience, gender, age, and type of work shift) predictors for the clinical competence and utilization of clinical competence, respectively (Table 5).

Based on the results presented in Table 5, in the first step, moral distress, as the strongest predictor variable, negatively predicted 17.9% of the variance in clinical competence (R^2^ = 0.179, P < 0.001) and 16% of the variance in utilization of clinical competence (R^2^ = 0.160, P < 0.001).

**Table 5:** Summery of multiple linear regression model (stepwise method)

Criterion variable	Model	Predictor	Beta	t	Sig.	R	R^2^	Adjusted R^2^
Clinical competence	1					0.430	0.185	0.179
		(Constant)		26.02	< 0.001			
		Moral distress	− 0.43	-5.86	< 0.001			
	2					0.463	0.214	0.204
		(Constant)		18.85	< 0.001			
		Moral distress	− 0.39	-5.21	< 0.001			
		COVID-19 ICU work experience	0.18	2.37	0.019			
	3					0.490	0.240	0.225
		(Constant)		17.12	< 0.001			
		Moral distress	− 0.39	-5.31	< 0.001			
		COVID-19 ICU work experience	0.17	2.31	0.022			
		Nursing work experience	0.16	2.28	0.024			
	4					0.510	0.260	0.240
		(Constant)		15.00	< 0.001			
		Moral distress	− 0.37	-5.19	< 0.001			
		COVID-19 ICU work experience	0.16	2.22	0.027			
		Nursing work experience	0.15	2.05	0.042			
		Gender (reference group: female)	− 0.14	-1.98	0.049			
Utilization of clinical competence	1					0.406	0.165	0.160
		(Constant)		26.31	< 0.001			
		Moral distress	− 0.41	-6.14	< 0.001			
	2					0.459	0.211	0.203
		(Constant)		19.41	< 0.001			
		Moral distress	− 0.36	-5.53	< 0.001			
		COVID-19 ICU work experience	− 0.22	3.34	0.001			
	3					0.496	0.246	0.234
		(Constant)		17.08	< 0.001			
		Moral distress	− 0.35	-5.59	< 0.001			
		COVID-19 ICU work experience	0.19	3.14	0.002			
		Gender (reference group: female)	− 0.18	-2.97	0.003			
	4					0.518	0.268	0.253
		(Constant)		10.68	< 0.001			
		Moral distress	− 0.37	-5.84	< 0.001			
		COVID-19 ICU work experience	0.18	2.93	0.004			
		Gender (reference group: female)	− 0.17	-2.83	0.005			
		Age	0.15	2.39	0.018			
	5					0.536	0.287	0.268
		(Constant)		4.50	< 0.001			
		Moral distress	− 0.33	-5.11	< 0.001			
		COVID-19 ICU work experience	0.19	3.09	0.002			
		Gender (reference group: female)	− 0.17	-2.74	0.007			
		Age	0.19	3.00	0.003			
		Type of work shift ((reference group: fixed)	0.15	2.22	0.027			

## Discussion

This study aimed at determining the relationship between moral distress and clinical competence of nurses working in COVID-19 ICUs.

The results demonstrated that moral distress in nurses working in COVID-19 ICUs is at a moderate level which was consistent with previous literature [53, 55, 56], although it contrary to the results of a research by Donkers et al. [35], and Arafat et al. [57]. The reason for this disparity may be attributed to the differences in the personal or organizational conditions. These results show the need for effective planning to reduce the moral distress of nurses, especially in critical situations, by health officials and policy makers.

In this study, it was found that most moral distress was related to the dimension of errors. Silverman et al. (2021) pointed out that during Coronavirus pandemic, due to the increased number of COVID-19 patients, to provide sufficient personnel, the immediate implementation of the team model (a combination of critical care nurses and moderate care with ICU nurses) can cause a lot of confusion for nurses and safety risks that would never happen in a regular ICU [58]. Organizational limitations, communication problems, death and deterioration of the patient’s condition, futile actions, malpractice and treatment-care errors are among the factors related to moral distress in nurses [59].

Moral distress is an inevitable byproduct of patient care that cannot be completely eliminated from the workplace; yet, there are strategies for adequate workplace resources, improved work life, and managerial and organizational leadership that organizations can use. They can apply it to reduce the negative effects of moral distress [18, 32, 60].

The findings further revealed that the clinical competence of the studied nurses and the application of nursing skills based on self-assessment were at a good and acceptable level. The clinical competence of Iranian nurses has been reported to be at the moderate to high levels [7, 61]. Moderate and high levels of clinical competence have also been reported in South Korean [2, 62], Lithuanian [63] and Taiwanese [64] nurses. The findings of another study that compared the clinical competence of nurses working in the COVID-19 ward and other clinical wards showed that in both groups, the average clinical competence was at an average level, and this average was significantly higher in nurses who worked in non-COVID-19 wards [65].

In the present study, the highest and lowest mean pertained to dimensions of the managerial abilities and the quality assurance, respectively. These findings are consistent with the results of the study by Kalantari et al. [66]. Yet, in Faraji et al.‘s study, the highest average score among the subscales of clinical competence was related to “situation management”; among the subscales of skills application, it was related to the subscale of “therapeutic interventions” [7]. In Arshadi et al.‘s study, the highest skill level pertained to the clinical skill of general knowledge of COVID-19 and the lowest skill level pertained to decision-making skills [61].

The difference in results could be attributed to participants’ different work sectors, data collection tools, or current work conditions related to COVID-19. In the current study, treatment measures that included planning, decision-making, and implementation of care based on the clinical situation and consultation with other employees had the lowest level. Of course, the large number of patients, lack of manpower, and high workload during the COVID-19 period can be the reason; this should be noticed by the authorities in times of crisis.

The findings of the study showed that there was an inverse and significant relationship between moral distress and its dimensions with clinical competence and skills application; this means that with increasing score of distress and its dimensions, the clinical competence of nurses and their skills application decrease. Moral distress was a significant negative predictor of clinical competence. Results from this study support those of previous studies that found an inverse relationship between job stress and clinical competence [44–46, 48, 50]. A key component of the moral distress is that a person is prevented from fulfilling a perceived obligation [67]. The findings of this study show that moral distress reduces clinical competence and its utilization in nursing practice. The COVID-19 pandemic poses ethical challenges for ICU professionals, potentially causing moral distress [35]. Feelings of powerlessness, anger, guilt, self-doubt, loss of self-esteem, frustration, and burnout are the consequences of moral distress that nurses may experience [68]. These consequences can be lasting [69], and have a negative effect on clinical competence and the application of these competencies.

Considering the relationship between moral distress and clinical competence, nursing managers can strengthen clinical competence and skills application by using strategies to deal with and reduce moral distress in nurses, especially in critical situations. To reduce moral distress, due attention ought to be paid to both individual and organizational factors [70]. Some factors are useful in reducing moral distress and thus improving professional competence. These include: educating nurses through holding ethics workshops, providing counseling for nurses, changing the working ward, revising procedures and instructions, reducing working hours, informing nurses of moral distress and its consequences, appropriate leadership practices, etc. [53].

### Limitations of the study

This study suffered from some limitations. One of the limitations was the use of a self-reporting tool, and there is a possibility of social desirability bias. There were confounding variables such as factors affecting nurses’ concentration that could not be controlled. A cross-sectional design was used in this research. For this reason, it makes causality impossible. Besides, since the present study was conducted on selected nurses of just one city, caution should be exercised in generalizing the results as this jeopardizes the external validity of the findings.

## Conclusion

The results of this study showed that there was an inverse and significant relationship between moral distress and each of its dimensions with the level of clinical competence and skills application. It is necessary for nursing managers to measure and control moral distress, especially in crises, to maintain the quality of nursing services in crises while maintaining the clinical competence of nursing.

## Data Availability

The datasets generated and analyzed during the current study are not publicly available due to an agreement with the participants on the confidentiality of the data but are available from the corresponding author on reasonable request.

## List of Abbreviations

MSc: Master of Science
BS: Bachelor of Science
SD: Standard Deviation
KS: Kolmogorov-Smirnov
ANOVA: Analysis of Variance
ICU: Intensive Care Unit
COVID-19: Coronavirus Disease 2019
S-CVI/Ave: Scale Content Validity Index/average
SMS: Short Message Service
